# Assessment of leaf morphological, physiological, chemical and stoichiometry functional traits for understanding the functioning of Himalayan temperate forest ecosystem

**DOI:** 10.1038/s41598-021-03235-6

**Published:** 2021-12-10

**Authors:** Monika Rawat, Kusum Arunachalam, Ayyandar Arunachalam, Juha M. Alatalo, Rajiv Pandey

**Affiliations:** 1 School of Environment and Natural Resources, Doon University, Dehradun, 248001 India; 2 Indian Council of Agricultural Research (ICAR), Krishi Bhawan, New Delhi, 110001 India; 3 Environmental Science Center, Qatar University, Doha, Qatar; 4 Indian Council of Forestry Research and Education, Dehradun, India

**Keywords:** Ecology, Ecology

## Abstract

Leaf functional traits support plant survival and growth in different stress and disturbed conditions and respond according to leaf habit. The present study examined 13 leaf traits (3 morphological, 3 chemical, 5 physiological, and 2 stoichiometry) of nine dominant forest tree species (3 coniferous, 3 deciduous broad-leaved, 3 evergreen broad-leafed) to understand the varied response of leaf habits. The hypothesis was to test if functional traits of the conifers, deciduous and evergreen differ significantly in the temperate forest and to determine the applicability of leaf economic theory *i.e.*, conservative vs. acquisitive resource investment, in the temperate Himalayan region. The attributes of the functional traits *i.e.,* leaf area (LA), specific leaf area (SLA), leaf dry matter content (LDMC), leaf water content (LWC), stomatal conductance (Gs), and transpiration (E) followed the order deciduous > evergreen > coniferous. Leaf carbon and leaf C/N ratio showed the opposite pattern, coniferous > evergreen > deciduous. Chlorophyll (Chl) and photosynthetic rate (A) were highest for evergreen species, followed by deciduous and coniferous species. Also, structural equation modelling determined that morphological factors were negatively related to physiological and positively with chemical factors. Nevertheless, physiological and chemical factors were positively related to each other. The physiological traits were mainly regulated by stomatal conductance (Gs) however the morphological traits were determined by LDMC. Stoichiometry traits, such as leaf C/N, were found to be positively related to leaf carbon, and leaf N/P was found to be positively related to leaf nitrogen. The result of the leaf functional traits relationship would lead to precise prediction for the functionality of the temperate forest ecosystem at the regional scale.

## Introduction

Leaf traits are morphological, physiological, chemical, phenological and stoichiometry characteristics influencing the growth, distribution, reproduction, and survival of the tree species^1–4^. Leaves regulate ecological processes, such as exchange of gases, carbon storage, photosynthesis assimilation, transpiration, respiration, nutrient cycling^2^. Leaf morphological traits, e.g., leaf area (LA) of a species determines canopy structure and surface availability for intercepting photosynthetically active radiation (PAR), and thus productivity in forest ecosystems^5,6^. Specific leaf area (SLA) determines the plant strategy of a species and provides information on photosynthetic capacity^7,8^. Leaf dry matter content (LDMC) explains plant growth rate and carbon assimilation^9^, while leaf nitrogen and phosphorus concentrations (LNC and LPC respectively) are good indicators of plant photosynthetic capacity and resource use strategy^1,10^. Similarly, photosynthetic rate (A), transpiration (E) and stomatal conductance (Gs) are indicators of CO_2_ and water exchanges. Therefore, knowledge of leaf morphological, chemical, physiological and stoichiometry traits can make a significant contribution to understanding the mechanisms of plant adaptation to future changes in environmental conditions^11,12^. Moreover, studying leaf functional traits at the regional scale is important for understanding trait-environment interaction at the global scale and develop predictive models. Therefore, unraveling the functional background of leaf traits can improve understanding of plant growth and its relationship with the environment^8^.

Changes in functional traits have significant impacts on the structure and composition of forests and the overall functioning of the ecosystem^13^. For example, functional trait changes provide support for trait-based habitat filtering in plant community^14^, species coexistence, and adaptive plant strategy^15–17^. However, the causes of high variability in leaf morphological and eco-physiological traits are due to different edaphic and climatic conditions, which assist in the coexistence and adaptation of the species in a forest ecosystem^18^. Moreover, biomass, carbon, and productivity of the forest are also dependent on the leaf traits^5^. Variation in the plant functional traits are regulated by the environmental constraints “*i.e.* climate, resource availability and disturbance level in the ecosystem^19^”. Moreover, trait-based modeling attempts to simulate ecological processes and requires detailed input parameters for ecosystem modelling^20^. The model-based information is useful for forest management and planning for ensuring the sustainable flow of ecosystem services.

Himalayan forests differ from other forests in terms of their climate, and are characterized by concentrated summer rainfall, mild winters, high sun angles, high elevation, and low annual variability in day length^21^. These conditions provide higher foliar biomass and nutrient sequestration, as well as higher net primary productivity to the forest ecosystem^18^. As a result, species found in the temperate ecosystem have particular and distinctive traits which allow them to function in these environmental conditions. Information regarding the temperate forests of the Himalayan region is limited, however, making it difficult for forest managers to understand the functioning of these forests. The lack of information prevents policymakers from formulating scientific-based forest management policies and thereby managing the degradation of the forests. With this in mind, we conducted trait variation evaluation in order to demonstrate the coexistence, adaptive strategy, and environmental constraints of the species in India’s temperate forests. Furthermore, the study was intended to facilitate better understanding of the functioning of the temperate forests, and to provide input for trait-based predictive modeling. The study attempted to evaluate variation among the traits (morphological, physiological, and chemical) of the conifers, deciduous broad-leaved, and evergreen broad-leaved tree species of the temperate forest, and to establish correlations between the functional traits of the tree species. We selected 13 leaf traits known to be indicators of plant morphological, physiological, and chemical status^22,23^, as well as important contributors to productivity, biomass production, decomposition, carbon storage, nitrogen utilization, and nutrient cycling^24^. We also analyzed the stoichiometry carbon/nitrogen ratio (C/N) and nitrogen/phosphorus ratio (N/P), as they can affect the environment^25^ and signal changes associated with the life history of plants. A complementary or alternative idea is that leaf economic theory may be applied to account for the functionality of temperate Himalayan forests, based on the trade-off strategy adopted by trees in exchanges such as conservative vs. acquisitive resource investment. Acquisitative species, which are characterized by high photosynthetic, respiration and growth rates, and short-lived and nitrogen-rich leaves, prefer nutrient-rich and/or growth-friendly environmental conditions. Conservative species, meanwhile, are those which display the opposite characteristics^1^. Leaf economic theory describes the relationships between a variety of leaf characteristics, such as carbon assimilation rate, leaf longevity, leaf mass-per-area, and nitrogen content^1^. The significance of leaf economic theory is that it can describe observed differences in plant strategies and indicate significant constraints on nutrient fluxes. Furthermore, leaf economic theory can be applied on a variety of scales, such as between species, within species, and even within individual plants^26^.

Our hypothesis is that functional traits of the conifers, deciduous broad-leaved and evergreen broad-leaved differ significantly in the temperate forest such as deciduous broad-leaved having highest values of SLA and lowest of LDMC, conifers having lowest values of SLA and highest of LDMC, and evergreen broad-leaved having intermediary values for both traits. These hypotheses were tested by evaluating the 13 selected key leaf functional traits in nine dominant tree species (3 coniferous, 3 deciduous broad-leaved, 3 evergreen broad-leafed) in a temperate forest in the Indian Himalayas. Trees were selected based on diameter at breast height (DBH 23.44 to 39.99 cm), to achieve uniformity amongst species in the same location. Morphological traits studied were: LA, SLA, LDMC; chemical traits: LCC, LNC, LPC; physiological traits: LWC, chlorophyll (Chl), A, Gs, E, and stoichiometry traits: leaf C/N and leaf N/P (Table 1). Productivity of the forest is dependent on these morphological, chemical, and physiological traits, and therefore variation in the traits can support in coexistence and adaptation of the species in a forest ecosystem. With this background, the main objectives of the study were to (a) quantify the variation in the 13 leaf traits among the different plant functional groups (conifer, deciduous broad-leafed, evergreen broad-leafed) and (b) applicability of leaf economic theory along with determination of the traits *i.e.* morphological, physiological, chemical and stoichiometry causal relationship for understanding the functionality of the temperate forest ecosystem. Structural Equation Modeling (SEM) was used to evaluate causal relationships among functional traits. Moreover, the information was also used to evaluate the applicability of leaf economic theory for the functionality of the temperate forests of the Himalayas.

**Table 1:** Classification of tree species in a temperate forest in Mussoorie Forest Division, Uttarakhand, Indian Himalayas.

Species	Family	Leaf habit	Leaf type	Tree density (trees ha^−1^)
*Abies pindrow,* Spach Ham	Pinaceae	Coniferous	Needle	220
*Cedrus deodara,* Loud	Pinaceae	Coniferous	Needle	250
*Pinus wallichiana,* Jackson	Pinaceae	Coniferous	Needle	210
*Aesculus indica,* Colebr	Hippocastanaceae	Deciduous	Broad	240
*Pyrus pashia,* Buch.Hemex D.Don	Rosaceae	Deciduous	Broad	160
*Toona ciliata,* R	Meliaceae	Deciduous	Broad	157
*Euonymus pendulous,* Wall	Celastraceae	Evergreen	Broad	123
*Quercus leucotrichophora,* A.Comm	Fagaceae	Evergreen	Broad	250
*Rhododendron arboreum,* Smith	Ericaceae	Evergreen	Broad	260

(*Abies pindrow* (40132), *Cedrus deodara* (70684), *Pinus wallichiana* (151787), *Aesculus indica* (50907), *Pyrus pashia* (49487), *Toona ciliata* (106761), *Euonymus pendulous* (51008), *Quercus leucotrichophora* (49548) and *Rhododendron arboreum* (69913)).

## Results

### Variations in morphological traits

**Figure 1:**
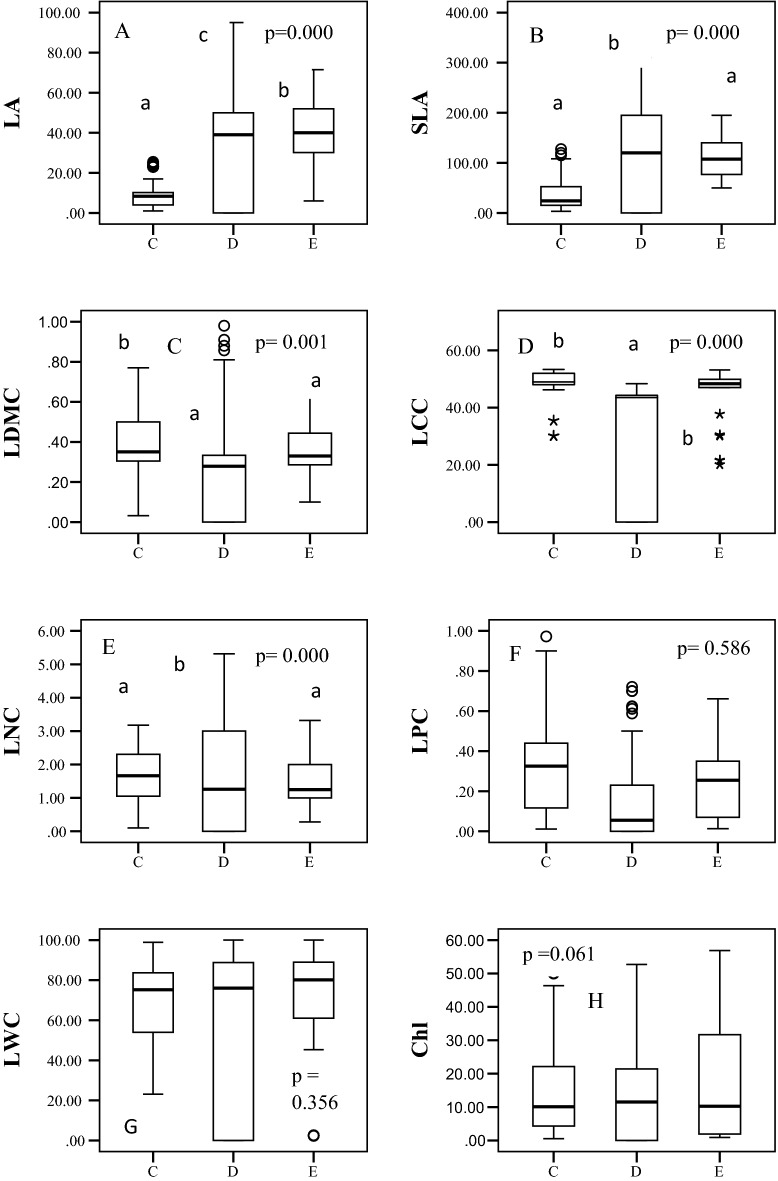

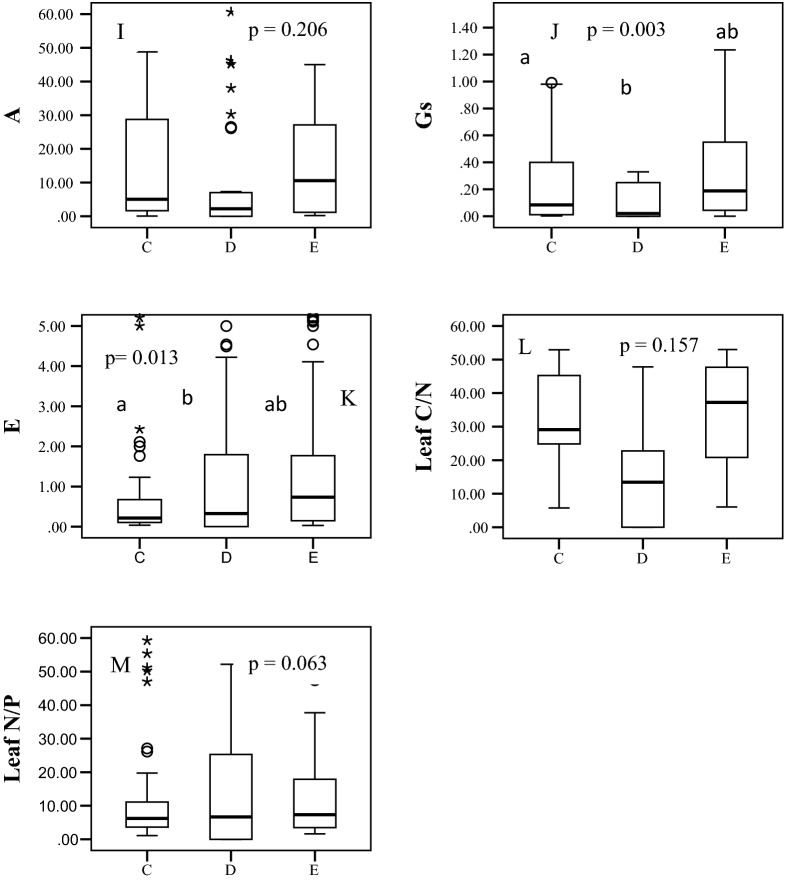
Distribution of values of various traits of tree species of different leaf habits in a temperate forest in Mussoorie Forest Division, Uttarakhand, Indian Himalayas (C = conifers, D = deciduous broad-leaved, E = evergreen broad-leaved): Morphological; LA = leaf area, cm^2^; SLA = specific leaf area, cm^2^ g^−1^; LDMC = leaf dry matter content, %; Chemical; LCC = leaf carbon content, %; LNC = leaf nitrogen content, %; LPC = leaf phosphorus content, %; Physiological; LWC = leaf water content, %; Chl = chlorophyll, mg g^−1^; A = photosynthetic rate, μmol CO_2_ m^−2^ s^−1^; Gs = stomatal conductance, mol H_2_O m^−2^ s^−1^; E = transpiration rate, mmol H_2_O m^−2^ s^−1^; Stoichiometry; Leaf C/N and Leaf N/P. The centerline indicates the median, upper and lower box heights indicate interquartile range. The alphabets within the figure represents results of post ANOVA multiple comparison by LSD. Different alphabets represents statistically different groups.

LA ranged from 34.00 to 173 cm^2^ for deciduous broad-leaved species, 6.00 to 71.50 cm^2^ for evergreens broad-leaved and 1.00 to 50 cm^2^ for conifers in the temperate forest studied (Fig. 1A). Mean SLA was 178.83, 112.00, and 43.31 cm^2^ g^−1^ in deciduous broad-leaved, evergreen broad-leaved, and coniferous tree species, respectively (Fig. 1B). However, LDMC was similar for conifers and evergreens broad-leaved (0.36%), but slightly higher for deciduous broad-leaved species (0.41%) (Fig. 1C). Therefore, LA and SLA were both maximum in deciduous broad-leaved species, followed by evergreen broad-leaved and coniferous species (Fig. 1A,B). LA and SLA differ significantly across the studied leaf habits, however, deciduous broad-leaved and evergreen broad-leaved were statistically similar and differ significantly with conifers for LDMC (Fig. 1A,B).

### Variations in chemical and stoichiometry traits

LCC ranged from 28.00 to 48.36% (mean 42.21%) in deciduous broad-leaved species, from 20 to 60% (mean 45%) in evergreen broad-leaved species, and from 30.12 to 86.44% (mean 52.17%) in coniferous tree species (Fig. 1D). Mean LNC was 2.50%, 1.60%, and 2.00% in deciduous broad-leaved, evergreen broad-leaved, and coniferous tree species, respectively (Fig. 1E). Differences in LCC and LNC were significantly different with leaf habits (Fig. 1D,E) and LPC was more or less similar in all leaf habits *i.e.* non-significant at 5% level (Fig. 1F). Conifer and evergreen broad-leaved species were homogeneous group for LCC and LNC, and statistically differ with deciduous broad-leaved (Fig. 1D,E). Differences in stoichiometry traits *i.e.* leaf C/N and leaf N/P were statistically non-significant *i.e.* invariant across the three leaf habits (Fig. 1L,M).

### Variations in physiological traits

LWC, Chl and A were insignificant among the three leaf habits (Fig. 1G–I). Chl was 21.70 mg g^−1^ in deciduous broad-leaved species, 18.12 mg g^−1^ in evergreens broad-leaved, and 13.16 mg g^−1^ in conifers (Fig. 1H). A ranged from 0.35 to 60 μmol CO_2_ m^−2^ s^−1^ (mean 15.42 μmol CO_2_ m^−2^ s^−1^) in deciduous broad-leaved species, from 0.23 to 81 μmol CO_2_ m^−2^ s^−1^ (mean 18.01 μmol CO_2_ m^−2^ s^−1^) in evergreens broad-leaved, and from 0.20 to 48.77 μmol CO_2_ m^−2^ s^−1^ (mean 14.93 μmol CO_2_ m^−2^ s^−1^) in conifers (Fig. 1I). Gs varied significantly between deciduous broad-leaved and conifers, but did not show significant variation between deciduous broad-leaved and evergreen broad-leaved, nor between evergreen broad-leaved and coniferous (Fig. 1J). E varied significantly among deciduous, evergreen broad-leaved, and conifers (Fig. 1K), while conifer and evergreen broad-leaved were homogeneous for E, as were deciduous broad-leaved and evergreen broad-leaved.

### Causal relationship among leaf morphological, chemical, and physiological traits

**Figure 2:**
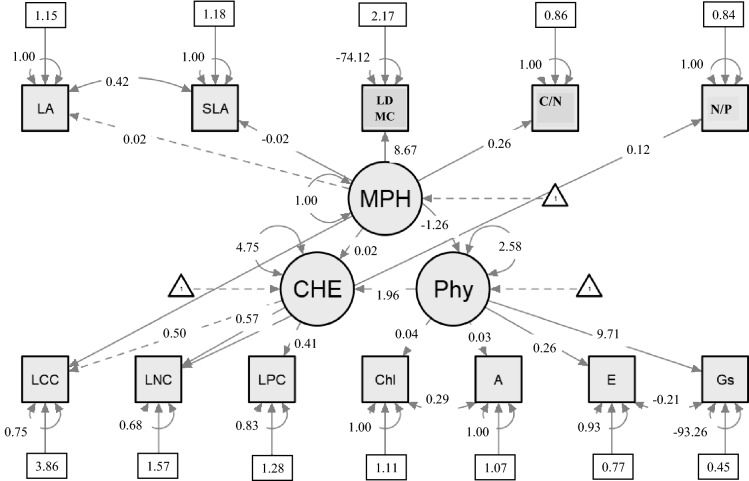
Structural Equation Modeling (SEM) with JASP (Jeffreys’s Amazing Statistics Program) software (A free and Open Lience) “SEM package” (JASP 0.14.1) were used for evaluation of causal relationship among leaf morphological, physiological, chemical and stoichiometry traits in the temperate forest ecosystem. Square nodes indicate manifest variables, circular nodes indicate latent variables, and triangular nodes indicate constant variables (intercepts). Directed edges (single-headed) indicate one variable having an effect on another variable *i.e.* linear regression parameters and bidirectional edges indicate (co)variances (correlation “r”) between two variables, and the circular curved arrows represent the variance of a variable. The path coefficients represent standardized partial regression coefficients. Dashed line indicates weak relationship, thicker line strong relationship and numbers in brackets are regression coefficients. Morphological; LA = leaf area, cm^2^; SLA = specific leaf area, cm^2^ g^−1^; LDMC = leaf dry matter content, %; Chemical; LCC = leaf carbon content, %; LNC = leaf nitrogen content, %; LPC = leaf phosphorus content, %; Physiological; LWC = leaf water content, %; Chl = chlorophyll, mg g^−1^; A = photosynthetic rate, μmol CO_2_ m^−2^ s^−1^; Gs = stomatal conductance, mol H_2_O m^−2^ s^−1^; E = transpiration rate, mmol H_2_O m^−2^ s^−1^; Stoichiometry; Leaf C/N and Leaf N/P. The R^2^ value for LCC, LNC, LPC, E, and Chl was 0.25; 0.32; 0.17, 0.07, and 0.02 with respective latent factor. The latent factors are morphological traits (MPH) (LA + SLA + LDMC), chemical traits (CHE) (LCC + LNC + LPC) and physiological (PHY) (Chl + A + E + Gs); and regressions are CHE vs MPH and PHY vs MPH and CHE; and the residual covariances were LDMC vs SLA and LCC vs LPC.

Structural equation modeling (SEM) was applied to establish causal relationships among leaf morphological, chemical, physiological and stoichiometry traits (χ^2^ = 286.43, d.f. = 46, p =  < 0.001, AIC = 10,613.33). SEM allowed identification of few significant causal relationships among leaf morphological, chemical, physiological, and stoichiometry traits (Fig. 2). Morphological traits were negatively (β = − 1.26) linked with physiological traits while positively with chemical traits. However physiological and chemical traits were positively related to each other (β = 1.96). Gs (β = 9.71) was having a strong direct effect followed by E (β = 0.26) for accounting the physiological features, with weak relationship for Chl (β = 0.04) and A (β = 0.03). The result indicated that with Gs increases, E decreases (r = − 0.21), however, Chl and A were positively (r = 0.29) related. LCC (β = 0.50), LNC (β = 0.57) and LPC (β = 0.41) were positively correlated with the functioning of the tree community. Morphological features were directly related to LDMC (β = 8.67) and LA, but indirectly related to SLA. Stoichiometry traits *i.e.*, leaf C/N and leaf N/P were related with leaf chemical traits *i.e.* leaf C/N was positively related (r = 0. 26) with LCC, and leaf N/P were positively related (r = 0.12) with LNC.

## Discussion

Evergreen leaves were characterized by higher leaf construction costs, slow nutrient returns, and tougher laminae. In contrast, deciduous leaves was associated with a higher photosynthetic rate per unit leaf mass, due to their higher LNC and SLA, higher intrinsic photosynthetic capacity, and less competition for light and carbon dioxide^27^. Evergreen broad-leaved favors infertile soils, longer leaf life span, and greater shade tolerance, that reduce seasonal variance in leaf exchange. In contrast, deciduous broad-leaved are favored by high seasonality, thermal, moisture, and light conditions, which are positively correlated across seasons^28^.

Morphological, chemical, physiological, and stoichiometry functional traits varied among different leaf habits in temperate forests and support for understanding the functioning of the forest ecosystem. In general, present evaluation observed variation in the traits and the order was: deciduous > evergreen > coniferous. However, LCC and leaf C/N ratio showed the reverse pattern (coniferous > evergreen > deciduous). There was little variation in LNC and LPC between the three leaf habits. Furthermore, present study found higher LA, SLA, and LDMC in deciduous broad-leaved species than in conifers and evergreen broad-leaved tree species. The higher LA and SLA in the deciduous broad-leaved species might be due to higher light interception^1,29^. Also, the higher SLA in deciduous broad-leaved species in tropical and subtropical forest indicates an acquisitive plant strategy^8,30^, while lower SLA in evergreens and conifers in subtropical forest indicates a conservative plant strategy^1,31^. LCC was significantly higher and LNC lower in conifers and evergreen broad-leaved plants in comparison to deciduous broad-leaved while LPC did not differ significantly among leaf-habits. A previous study on global data synthesis reported that leaves of conifers and evergreen broad-leaved plants have a higher carbon than deciduous broad-leaved species^32^. Higher carbon may be attributed to the presence of higher lignin content in conifers in comparison to the deciduous^33^. Leaf C/N was highest for conifers; however, leaf N/P was highest for deciduous species. The leaf C/N was high than subtropical forests^8,34^, possibly due to the high absorption capacity and utilize efficiency of nitrogen in the subtropical region^8^. High LWC is also reported for deciduous trees in temperate and boreal forests^35,36^. Chl and A were highest for evergreen broad-leaved tree species, possibly due to the higher availability of light and interaction with LNC. The availability of the leaf throughout the year enables the evergreen to use the nutrients to support the new growth and control photosynthesis^18^. Gs and E of leaves were lowest in deciduous species. The variation in the leave traits might be due to a robust leaf structure of evergreen species, which resists CO_2_ diffusion resulting in lower mesophyll conductance such as Gs^37–40^ and E^41^.

Our analysis for the evaluation of variations in leaf functional traits was across woody species in a temperate forest in the Indian Himalayan region. Among the different types of species studied, the three conifer species (*Abies pindrow*, *Cedrus deodara*, and *Pinus wallichiana*) had higher LCC and LDMC and are thus said to follow resource conservation strategy^42^. The three broad-leaved deciduous species (*Aesculus indica*, *Pyrus pashia*, and *Toona ciliata*) had higher SLA and LNC, indicating resource acquisition strategy. The three broad-leaved evergreen species (*Euonymus pendulous*, *Quercus leucotrichophora*, and *Rhododendron arboretum*) had high leaf Chl and A, and the traits leaf Chl and A did not exhibit significant differences among the three types. Although this study was limited to a specific region and climate, we do believe the results can be extended to other temperate forests dominated by the same representative species.

According to leaf economic theory coniferous have a resource conservation strategy with low SLA while the deciduous, have a strategy of fast acquisition with high SLA. Moreover, less productive plants tend to have low SLA, high LDMC, and leaves with long longevity (resource conservation strategy) however in the productive environment, the plants tend to have high growth rates, high SLA, low LDMC, low longevity leaves (fast acquisition strategy). The results of our study support the predictions of leaf economic theory, which is useful for understanding of functioning and a tool for predicting the responses of the forest vegetation to environmental changes, as plant strategies are dependent on the interactions among multiple traits^1^. This study provide information for further understanding the mechanism of species coexistence and predicting which kinds of species may assemble in a particular region in response to changes in environmental conditions.

We also observed that among all the morphological traits, LDMC was having strong relationship as observed by others^7,8^. LA and SLA were corelated as LA is directly affects the SLA^43^. Among physiological traits, Gs was having the strongest relationship, probablly due to the increase in CO_2_ concentration in the atmosphere as observed by others^38,40,44^. We found a negative relationship between Gs and E that might be due to the rise in CO_2_ level^45,46^. Our study also observed the positive relationship between Chl and A in the temperate region as reported by others^36^.

## Conclusion

In the temperate forest studied, plant functional type classification explains forest ecosystem functioning. There have been few systematic studies on functional traits in temperate forest tree communities of the Himalayan region. This study investigated leaf functional traits concerning the morphology, physiology, chemical and stoichiometry component of the temperate forest tree community in this region. The results demonstrated that LA, SLA, LDMC, Gs, and E differed significantly between leaf habits, and their values followed the order deciduous > evergreen > conifers. The LCC showed the opposite pattern, conifers > evergreen > deciduous. Leaf Chl and A rates were highest for evergreen species, followed by deciduous and coniferous species. Overall, the variation in leaf functional traits affected leaf functions. Hence, species co-habiting in the same environment employ different plant adaptive strategies, *i.e.* conservative and acquisitive, for dealing with that environment. Moreover, variation in the functional traits among three-leaf habits largely supports the predictions of leaf economic theory.

## Material and methods

### Study site

The temperate forest studied lies in the Indian Himalayan region of India, in Mussoorie Forest Division, Uttarakhand (30°28′02.6″N, 78°05′47.9″E; 2277 m asl). The mean annual rainfall in the region is around 2200 mm and the mean annual temperature is 20 °C. The region experiences three main seasons, winter (October to February), summer (March to June), and rainy (July to September)^47^. Soils of the region are Leptosols, Regosols, and Cambisols, developed mostly on dolomite^48^. The natural vegetation of the area is predominantly dense mixed forest (evergreen, deciduous, and coniferous tree species). Weather patterns differentiating this temperate forest and the region’s geographical features have enabled dominance of species such as oak, rhododendron, and conifers^49^.

### Plant functional trait measurement

A vegetation survey was carried out using the quadrat method, where 20 quadrats, each measuring 10 m × 10 m, were laid out in the forest to study tree characteristics. The tree species were grouped into needle-leaved conifers, broad-leaved evergreens, and deciduous angiosperms (Table 1). LA, SLA, LDMC, LWC, and Chl were estimated based on measurements on five fresh, mature, fully expanded, and healthy leaves in five individuals per species, as described elsewhere^23^ (Table 2). LCC, LNC, and LPC were measured on fresh leaves collected from the forest, dried in the laboratory, crushed, and analyzed according to methods listed in Table 2. Leaf physiological traits, *i.e.*, A, E, and Gs were measured by LICOR XT-6400 photosynthesis equipment. The youngest and fully expanded leaves were used preferentially for measuring physiological parameters and measurements were made between 9 am and 2 pm under clear-sky conditions. A total of 45 observations were made for each parameter, on nine trees (five replicates per tree) (Table 2). Permissions were granted from the Uttarakhand Forest Department for the collection of plant, wood, and soil samples and authors followed the guidelines with the IUCN Policy Statement on Research Involving Species at Risk of Extinction and the Convention on the Trade in Endangered Species of Wild Fauna and Flora. Dr Pravin Kumar Verma, Scientist, Division of Forest Botany, Forest Research Institute, Dehradun identified the plant species. The name of herbarium used for identification was DD Herbarium, Forest Research Institute, Dehradun. The specimens of the species were authenticated with the already available vouchers in the DD Herbarium of FRI Dehradun.

**Table 2:** Leaf traits, abbreviations (Abb), units, measurement method/equation, and strategy in a temperate forest in Mussoorie Forest Division, Uttarakhand, Indian Himalayas.

Traits	Abb	Unit	Category	Measurement method/equation	Strategy
Leaf area	LA	cm^2^	Morphology	Leaf area meter (LI-3100C)	Resource capture
Specific leaf area	SLA	cm^2^ g^−1^	Morphology	$SLA=\frac{LA}{Leafdry \; weight \;(LDW)}$	Resource capture
Leaf dry matter content	LDMC	%	Morphology	$LDMC=\frac{LDW}{Leaf \; fresh \; weight \; (LFW)}$	Resource capture
Leaf carbon content	LCC	%	Chemical	CHNS (Euro EA-3000)	Nutrient
Leaf nitrogen content	LNC	%	Chemical	CHNS (Euro EA-3000)	Nutrient
Leaf phosphorus content	LPC	%	Chemical	Acid digestion method*	Nutrient
Leaf water content	LWC	%	Physiological	$LWC=\frac{LFW-LDW}{Leaf \; saturated \; weight-LDW}*100$	Water exchange
Chlorophyll	Chl	mg g^−1^	Physiological	UV Spectrophotometer	Food production
Photosynthesis	A	μmol CO_2_ m^−2^ s^−1^	Physiological	LICOR-6400XT	Gas exchange
Stomatal conductance	Gs	mol H_2_O m^−2^ s^−1^	Physiological	LICOR-6400XT	Gas exchange
Transpiration rate	E	mmol H_2_O m^−2^ s^−1^	Physiological	LICOR-6400XT	Gas exchange
Leaf carbon/nitrogen	C/N	Unit less	Stoichiometry		Nutrient limits
Leaf nitrogen/phosphorous	N/P	Unit less	Stoichiometry		Nutrient limits

*Species and their Voucher IDs used for identification.

### Data analysis

The mean values of all leaf traits was estimated by SPSS 23. The plant species are divided into three functional groups or leaf habits namely conifers, deciduous broad-leaved, and evergreen broad-leaved. ANOVA was applied to test the difference among the three functional groups for each functional trait and reported with Box-plot. Structural equation modeling was applied to examine the casual relationships among leaf morphological, physiological, chemical, and stoichiometric traits. SEM was conducted by the “lavaan package” and models were visualized with JASP software “SEM package”^38^.

### Theoretical settings: a priori model

**Figure 3:**
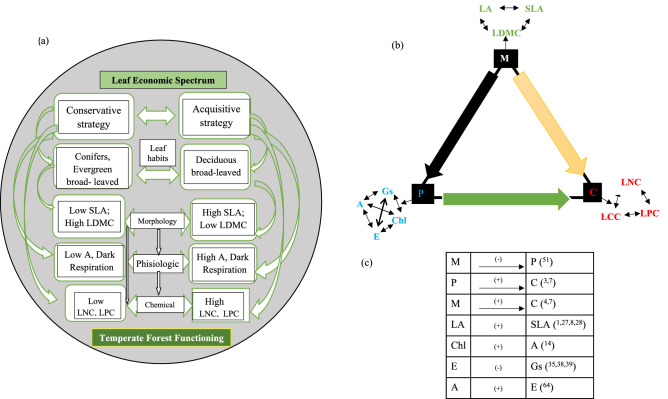
(**a**) Leaf economic spectrum of different leaf habits (**b**) Theoretical framework of the casual relationships among morphological, physiological, chemical and stochiometry traits (Symbols; M: Morphology, P: Physiology, C: Chemical) of temperate forest ecosystem and (**c**) evidences for relationship along with direction between traits (Some other proposed relationship was hypothesized to exist in temperate forest).

A theoretical framing of causal relationships between leaf morphological, physiological and chemical traits of temperate forest ecosystems, is explained in Fig. 3. According to an a priori model for the temperate forests, morphological and chemical features are understood to have a favorable relationship, while physiological traits and morphology are understood to have a negative relationship. This negative relationship may be attributed to the fact that many physiological processes are more plastic than structural processes^50^. Indeed, the physiology of trees can change dramatically without morphological changes in the short term^50^. Moreover, the difference in plasticity between morphological and physiological features is species-specific. Shade-intolerant species, for example, have greater physiological plasticity, while shade-tolerant species have greater morphological plasticity^51^. These complexities among the physiological, morphological and chemical features were used to assess species habitat affinities^50^, where leaf area was found to be positively associated with SLA^1,8,28^, as SLA influences canopy expansion and growth by changing total leaf area per plant and thereby affecting light interception and efficiency^52^. Chl, meanwhile, has a positive relationship with A^14^, as chloroplasts acclimatize to the environment and modulate the stoichiometry of components as per the requirements of plants^53^. A has a positive relationship with E^51^ for the management of leaf temperature^54,55^, and a negative relationship with Gs^35,38^. The stomata alter the aperture in response to external conditions in order to maximize the photosynthesis-water loss tradeoff^56^, and are therefore strategically linked to Chl and A. The responsiveness of leaf functional features to environmental variables, *i.e.* light and nutrient availability, is used by plant species to occupy environmental niches^57^. SLA is positively correlated with the relative growth rate of plants^58^, and reflects the potential rate of return on investment for a leaf intercepting light^59^. Leaf size accounts for water use efficiency and the amount of light intercepted for photosynthesis^59^, whereby SLA is positively correlated with the relative growth rate of plants^58,60^. Finally, the photosynthetic capacity of the leaves is positively associated with foliar N concentrations and specific leaf area^61^. Here we emphasize the significance of the ecological scale at which trait variation is considered, and suggests that common trait-by-trait scaling interactions should be handled with caution at regional to local scales. More specifically, PFT can be a useful predictor variable for inferring one feature from another. The results have significant implications for dynamic vegetation models at the local scale, and for using trait-based techniques to predict forest function at regional and local levels, depending on the availability of the data^62,63^.
